# Retroperitoneal schwannoma sandwiched between abdominal aorta and inferior vena cava. A case report

**DOI:** 10.1016/j.ijscr.2020.06.087

**Published:** 2020-06-24

**Authors:** Pramod Nepal, Yuko Mataki, Kosei Maemura, Hiroshi Kurahara, Kiyonori Tanoue, Yota Kawasaki, Tetsuya Idichi, Yuto Hozaka, Satoshi Iino, Shinichiro Mori, Hiroyuki Shinchi, Shoji Natsugoe

**Affiliations:** a Department of Digestive Surgery, Breast and Thyroid Surgery, Graduate School of Medicine, Kagoshima University, Sakuragaoka 8-35-1, Kagoshima, 890-8520, Japan; b Department of Health Science, Graduate School of Medicine, Kagoshima University, Sakuragaoka 8-35-1, Kagoshima, 890-8520, Japan

**Keywords:** CT, computed tomography, MRI, magnetic resonance imaging, EUS, endoscopic ultrasound, FNA, fine needle aspiration, IVC, inferior vena cava, Case report, Preoperative diagnosis of schwannoma, Retroperitoneal schwannoma

## Abstract

**Introduction:**

Schwannomas are tumors that originate from the Schwann cells present in the nerve sheath of peripheral nerves. They are commonly seen in cephalocervical areas. Schwannomas in the abdominal cavity are rare. Here, we discuss a case of retroperitoneal schwannoma lying dorsal to pancreas with critical relations to surrounding vessels.

**Presentation of case:**

A 74 years old asymptomatic male was found with elevated amylase level on his routine blood examination. MR imaging revealed retroperitoneal mass of size 21*18*24 mm. EUS-FNA confirmed retroperitoneal schwannoma. The patient had co-morbid renal disease and was on hemodialysis. During the latest follow up, the tumor was 41*37*41 mm in size located dorsal to the confluence of right renal vein and inferior vena cava. The tumor was in contact with inferior vena cava, horizontal part of duodenum, right renal artery, right kidney, and adrenal gland. The patient underwent laparotomy and the tumor was extract with intact capsule. There were no post-operative complications.

**Discussion:**

Pre-operative diagnosis in retroperitoneal schwannomas is challenging because imaging features are usually non-specific, and biopsy is the only diagnostic technique. EUS-FNA, which has low diagnostic accuracy, is useful in pre-operative diagnosis of small tumors devoid of intra-tumoral degeneration.

**Conclusion:**

Retroperitoneal schwannoma is a rare entity. Preoperative diagnosis and curative resection are technically difficult. Care should be given during preoperative investigations and surgical resection of the tumor. EUS-FNA can be a useful diagnostic tool.

## Highlights

• Retroperitoneal schwannomas in the abdominal cavity are rare and pose difficulty in preoperative diagnosis and management.
• Imaging modalities are usually not sufficient to conclude a diagnosis.
• EUS-FNA is useful to confirm preoperative diagnosis.
• In EUS-FNA, diagnosis can be accurate if the tumor size is small and tumor is devoid of intratumoral degenerations.

## 1 Introduction

Schwannomas are tumors that arise from the nerve sheath, commonly in head, neck, and extremities. They rarely manifest in the retroperitoneal area [1]. Approximately, 0.3–3 % of schwannomas are found in the retroperitoneum [2]. They are mostly benign, and the malignant cases are rare. Retroperitoneum is the second most common location for occurrence of sarcomas, and it is important to distinguish them from schwannomas so that the patients can be managed conservatively under surveillance [3]. The imaging techniques (ultrasound, computed tomogram (CT) abdomen, magnetic resonance imaging (MRI) and PET-CT abdomen) and histologic tests may help in pre-operative diagnosis but are not always conclusive [4]. Likewise, schwannomas at the time of surgery, are generally large (10–20 cm in diameter) because the patients are either asymptomatic or report vague abdominal symptoms [5]. Thus, they pose diagnostic and therapeutic difficulty.

In this report, we discuss a case of retroperitoneal Schwannoma, behind the head of pancreas and sandwiched between inferior vena cava (IVC) and abdominal aorta, which was operated in our University hospital. EUS-FNA is particularly important to confirm preoperative diagnosis, so that patient can be managed conservatively under supervision.

## 2 Presentation of case

**Fig. 1:**
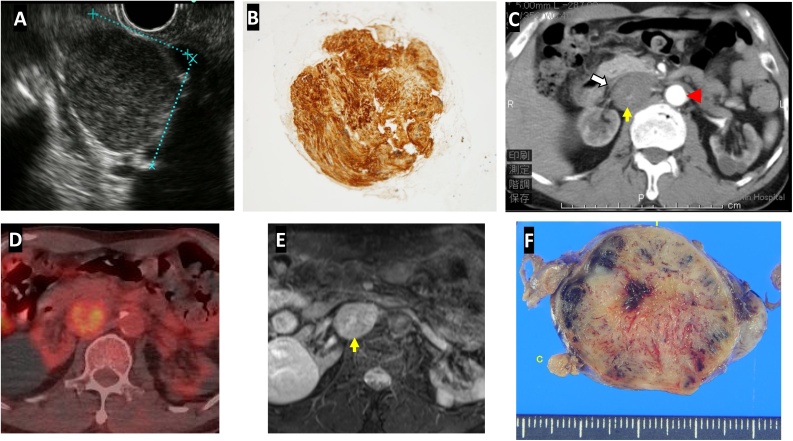
Preoperative investigations. A and B: EUS image at the time of diagnosis of tumor, and fine needle aspirate showing spindle shaped cells with S-100 staining C: Recent CT image of the tumor (yellow arrow) displacing IVC (white arrow) and sandwiched between IVC and abdominal aorta (arrowhead). D: T2-weighted MR image with intratumoral hyperintensity. E: Increased uptake in FDG-PET scan. F: The cut of resected specimen.

A 74 years old asymptomatic male patient was found with elevated amylase level on his routine blood examination. He was hypertensive and had a history of gastric cancer in a first degree relative. His physical and systemic examinations were unremarkable. On MRI, retroperitoneal mass of size 21*18*24 mm was spotted which revealed hypo intensity in T1 and hyperintensity in T2 weighted images, respectively. The tumor gradually enhanced from center to the periphery. Endoscopic ultrasonogram (EUS) revealed low echoic mass of size 24*21 mm located behind portal vein (Fig. 1A), and tissue aspirate extracted by 22-gauge needle via duodenum showed the follicular nests of spindle shaped tumor cells in a palisading pattern. The nuclei were round to spindle-shaped and pointed at both ends. The tumor cells positively stained for S-100 (Fig. 1B). At the same time, patient was diagnosed with chronic kidney disease due to nephrosclerosis, and the patient had gradual deterioration of renal functions ultimately needing hemodialysis support. The patient was kept under observation. During the latest follow up, the contrast enhanced CT abdomen showed a mass of 41*37*41 mm with clear margins located dorsal to the confluence of right renal vein and IVC. The tumor was in contact with IVC, horizontal part of duodenum, right renal artery, right kidney and adrenal gland (Fig. 1C). It was sandwiched between abdominal aorta and the IVC, displacing the later. MRI confirmed a retroperitoneal mass with mildly elevated intratumoral signal intensity in T2 weighted images and PET CT also showed slight signal uptake (Fig. 1D and E). The tumor doubling time was 653 days. We planned for surgical removal of the tumor.

**Fig. 2:**
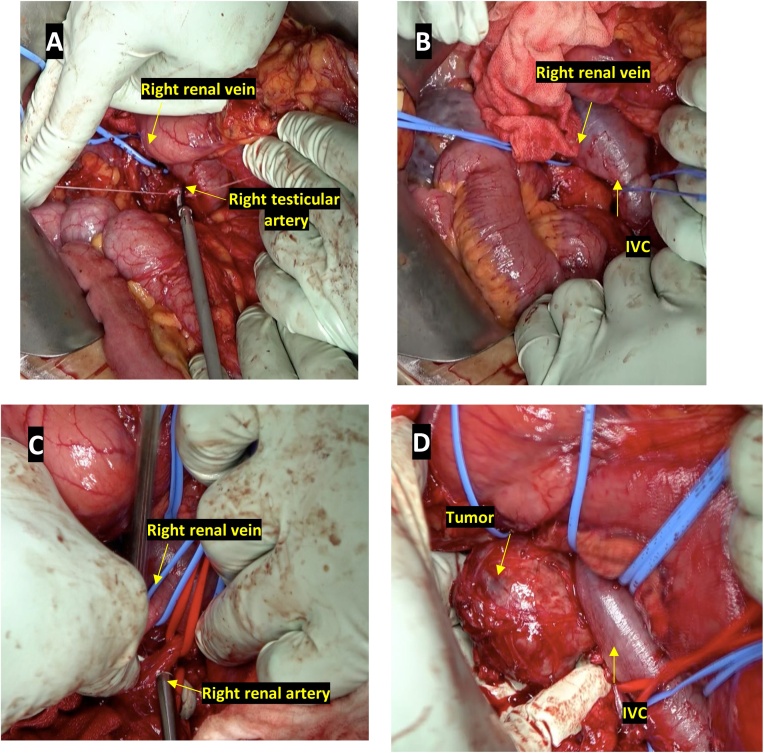
A. Right renal vein was taped, and right testicular artery was ligated. B. IVC and right renal vein were taped. C. Right renal artery and vein were preserved and inspected for sufficiency of blood flow. D. The dorsal caudal and cranial aspects of tumor was dissected, and tumor was peeled off en bloc.

Surgery was performed by a certified general surgeon with 16 years of experience. Patient was kept in supine position, and midline incision was given. Kocher maneuver was carried out; peritoneum was incised at the right edge of duodenum and dissection along the undersurface of duodenum was carried out while lifting and retracting it towards left. Intra-operative sonogram was used to verify tumor and its surrounding relations. Dissection was done on the right side of IVC and right renal vein was taped. The right testicular vein was clipped and divided (Fig. 2A). Likewise, dissection was done on the left side of IVC followed by taping of IVC (Fig. 2B). Tumor lied posterior to the IVC behind the confluence of right and left renal veins without any adhesions. Right renal artery, which was displaced by the tumor caudally, was isolated from it and the blood flow was inspected with blood flow meter (Fig. 2C). After that, the tumor was separated from IVC, dissected on its posterior aspect and peeled off (Fig. 2D). There were no intraoperative and immediate post-operative complications. The duration of surgery was 255 min and total blood loss was 240 mL. The cut of specimen revealed nonhomogeneous solid tumor with capsule (Fig. 1F). The patient was discharged on 12th post-operative day and kept under regular follow-up. There were no signs of recurrence and patient is enjoying a good quality of life.

This article is reported in accordance to SCARE reporting guidelines [6].

## 3 Discussion

Schwannomas are tumors of mesenchymal origin that originate from the Schwann cells present in the nerve sheath of peripheral nerves. They are mostly solitary, slow growing, homogenous and benign on nature. They may occur in any nerve trunks cranially and peripherally, except the cranial nerve I and II [2]. Schwannomas consist of 1% of all retroperitoneal tumors [3]. Malignant transformation is rare and is associated with von Recklinghausen’s disease [7]. Patients are usually asymptomatic. Diagnosis is made when the mass grows to a significant size and presents with non-specific or obstructive symptoms or during radiographic imaging for unrelated symptoms [8]. The symptoms are likely due to gastrointestinal and urinary organ displacement in the retroperitoneal space [5,8]. Also, schwannomas may erode overlying mucosa leading to bleeding or bowel obstruction [9].

Pre-operative diagnosis of retroperitoneal schwannomas is challenging. Cross-sectional radiology and core needle biopsy can differentiate the tumors from other malignant or invasive conditions [5]. On imaging, schwannomas are well-circumscribed mass with smooth, regular margins and without features of local invasion. It often has central low attenuation, peripheral enhancement, and cystic degeneration [9]. The MRI characteristics of benign retroperitoneal schwannomas include hypo intensity on T1 weighted and hyperintensity on T2 weighted imaging [10]. In MRI, target sign (peripheral hyperintensity with low central area) and fascicular sign (small ring like structures with peripheral hyperintensity that represents fascicular bundles within nerves) are reported [11]. In FDG-PET scan, schwannomas show wide range of uptake values, even in benign cases, because of various cell densities [12]. Although the imaging helps in determining size, margin, vascularity, and adjacent organ involvement of the tumor, its findings are non-specific and often not sufficient to conclude a diagnosis. There is no non-histological definitive diagnostic method. The imaging features mentioned above arise after intratumoral degenerative changes (cystic degeneration, hemorrhages, and calcifications) [8,9]. These features are particularly absent in small tumors, making them difficult to diagnose. The biopsy, which reveals hypercellular Antoni A and hypocellular Antoni B areas positive for s-100 and vimentin staining and negative for CD34, almost confirms the diagnosis but only if the sample contains enough Schwann cells to visualize microscopically [[13], [14], [15]]. It is reported that larger schwannomas tend to have undergone degenerations, and biopsy from those areas may be misinterpreted as malignancy [15,16]. Surgery is the only curative management. However, controversy exists over attaining negative margins [8,16]. Some surgeons recommend en bloc excision with negative margin because malignancy cannot be accurately ruled out in pre-operative biopsy or even during intra-operative frozen section [4]; while other group of surgeons recommend simple enucleation/ partial excision without extensive resection of adjacent tissue [2,7]. The surgical approach can be either open or laparoscopic [17]. The idea is to minimize post-operative complications by avoiding resection of adjacent structures when possible. In our case, we ruled out von Recklinghausen's disease as the patient had no relevant family history, Café au lait macules, characteristic body type such as short height, bigger head, and bony dysplasia. The closest differential diagnosis was leiomyosarcoma, which is unencapsulated and negative for S-100 protein. Diagnosis was confirmed with EUS-FNA. Although, EUS-FNA has low diagnostic accuracy, we recommend it as an important pre-operative diagnostic technique for small-sized schwannomas. Such tumors are usually devoid of intratumoral degenerations, and EUS-FNA can extract enough sample to confirm the diagnosis. In this case, because of preoperative confirmation made possible by EUS-FNA and small size of tumor, we could keep the patient under surveillance for six years and perform surgery when the tumor became large and patient’s renal condition was stable under hemodialysis. We opted for open approach considering the critical location of tumor adjacent to major vascular structures and patient’s co-morbid status.

## 4 Conclusion

Careful preoperative assessment should be carried out in patients with retroperitoneal masses. Preoperative diagnosis by imaging and curative resection are technically difficult. Although EUS-FNA has low diagnostic accuracy, it is especially useful for preoperative confirmation of retroperitoneal tumors and aids to formulate treatment strategy.

## Declaration of Competing Interest

We declare no conflict of interests.

## Funding

The authors did not receive any specific grant from funding agencies in the public, commercial, or not-for-profit sectors.

## Ethical approval

Not applicable.

## Consent

Consent to participate was taken.

## Author contribution

PN and YM conceived and designed the study and were involved in data collation. KM, HK, KT, SI, YK, YH, TI, HS and SM participated in designing the study, coordination, and data analysis. PN drafted the manuscript. SN participated in manuscript preparation and critical revision. All authors have read and approved the manuscript.

## Registration of research studies

1 Name of the registry:
2 Unique identifying number or registration ID:
3 Hyperlink to your specific registration (must be publicly accessible and will be checked):

## Guarantor

Yuko Mataki.

## Provenance and peer review

Not commissioned, externally peer reviewed.
